# Baltimore Gynæcological and Obstetrical Society

**Published:** 1888-02

**Authors:** 


### BALTIMORE GYNÆCOLOGICAL AND OBSTETRICAL SOCIETY.

### A CASE OF TETANOID CONSTRICTION OF THE UTERUS.

H. M. Wilson, M. D., said: Four years since, I was first consulted by Mrs. J., a lady of about 24 years of age, just married. She was of fine physique and healthy appearance. Her trouble at that time was uterine neuralgia. Several years before she had had stricture of the bowel, requiring surgical assistance. Nothing of special note occurred during the two years following, except a small subcutaneous abcess in the right iliac fossa, which was lanced, and soon healed. This reappeared in a smaller form — about the size of an almond — two months since. At the end of three years she became pregnant. Her gestation was accomplished without special distress, except on several occasions after moderate exercise she had slight attacks of syncope — the last, about four days prior to her confinement. Five days before labor, whilst in bed and without any exertion on her part, her water was discharged. I was called to see her at 6 o'clock in the morning, and found her bright and cheery, free of headache, with pains, described as sharp, at intervals of about ten minutes. Upon examination the soft parts were found to be in much better condition than had been feared. Bowels twice moved, kidneys acting freely. The os was entirely closed. Upon my return in two hours the os had opened to the size of a dime, edges soft and thin, and the head presented in the right occipito-anterior plane. From this time I remained with her. Quinine was ordered, to assist in relaxation. My patient now commenced to complain of nausea, to the extent of emesis, which was frequently, but not inordinately, repeated. The further progress was slow, but not requiring, in my judgment, either bleeding or medicine. At 5.30 in the afternoon, I deemed the os sufficiently dilatable to admit a careful application of the forceps (Simpson's), the head being within easy reach. No difficulty was encountered in their adjustment, but in the effort to deliver no advance could be made. Thinking the funis might be abnormally short or tightly wrapped about the neck, I substituted the Tarnier instrument. I could cause the caput to appear at the vulva so as to be seen, but instantly it would disengage itself from the blades and fly back to its former position. At this point I first observed her pallor, and, putting my finger on her pulse, was shocked to find it a mere thread. Instantly suspending the anæsthetic, I administered brandy freely by the mouth and in repeated hypodermics. The lady did not rally, and died within thirty minutes. We satisfied ourselves of the death of the fœtus, but, seeking for some solution of this terrible and inexplicable result, Dr. Miltenberger, at my request, effected the post-mortem delivery. Upon introducing his hand he found a band embracing the shoulders, which, to use his own words, "gave the impression of a band of steel." It was only after a prolonged and most persistent effort that version was finally accomplished.

Such were the different phases of the case.

Let us consider the remedial appliances; and first, as to anæsthetics: Chloroform was used occasionally, and in small quantities, for an hour probably, just at the completion of a pain, "to blunt its edge," as the lady expressed it, but not carried to its full effect; indeed, more as a placebo. This was thought proper on account of the lady's highly sensitive organism, and to quiet, as far as possible, her urgent demands for relief. When instruments were used it was inhaled freely, but, as I have before stated, instantly abandoned upon the appearance of dangerous symptoms. She came from under its influence speedily, asking questions, complaining of the strength of the brandy, demanding water, requesting to be fanned, etc., her intellect remaining unclouded to the last.

An accoucheur of large experience in a neighboring city wrote me of a case in his own practice very similar. He had supplemented what I had done by copious blood-letting, had failed with forceps, and after so long a time had succeeded in introducing his hand, but suffered the further complication of a ruptured uterus, losing both mother and child.

As to the immediate cause of death: In this case the lady passed from the influence of the chloroform quickly, and, so far as I could observe, entirely. She asked and answered questions, and her intellect was unclouded. As for the forceps, they were applied without difficulty and used with discretion. In nothing did the manipulation differ from their constant and ordinary use.

We must look for the real cause, I think, in the tetanoid condition of the uterus, as I have ventured to name it. Whether the opinion held by some be correct, that this condition is induced by the early evacuation of the waters, or that the source of danger must be sought elsewhere, I cannot determine. The reflex symptoms indicated a tetanic condition of the womb. Curiously enough, the lexicographers and surgeons speak of puerperal tetanus, but little or no mention is made of such a malady by any obstetrical author within my knowledge. Dr. R. P. Harris, in a note in Playfair, writes of "tetanoid falciform contraction of the uterus." Dr. Thomas C. Smith, in the "American Journal of Obstetrics," under the caption of "Ante-partum Hourglass Contraction of the Uterus," has given the fullest account I have seen of this form of dystochia. He has collated many reported cases as illustrating the general subject, referring to Dr. Roper's paper before the London Medical Congress in 1881. Dr. Hosmer's case, reported in the "Boston Medical and Surgical Journal," 1878, in which craniotomy was done, but without success, and a case reported by Dr. McDonald before the Edinburgh Obstetrical Society, 1878–79. But the nearest approach to the case under consideration is reported in the "Philadelphia Medical Recorder," 1821, by Dr. I. Baltzell, of Frederick, Md. After a fruitless trial of every expedient, the patient died undelivered, and upon a post-mortem examination the tetanic band was still so rigid as to require the knife in order to release the child.

How should such a case be treated? If the actual touch verifies the condition, the best procedure, in my opinion, would be version if possible, and in the failure of this the Cæsarean section. Should collaspe supervene, the free use of brandy, with external heat and electricity. Should its invasion be as rapid in point of time and progress as in my case, and in which respect it differs from all others known to me, then the prospect of success by the expedients suggested, or by any other, would seem to be very slight.

### LAPAROTOMY, WITH REPORT OF THREE CASES

H. P. C. Wilson, M. D., said: I present to the Society this evening the report of my last three cases of Laparotomy.

The first was for the removal of a compound multilocular tumor of the ovary.

The second, for the removal of a papillomatous tumor of the ovary, with abdominal dropsy.

The third, for the removal of both ovaries and tubes, in a woman to whom all other means of treatment for over sixteen years had failed to give relief, and a shattered mind and body had brought her to great desperation.

Within the last few years so much has been done and written in abdominal surgery that I shall not be expected to add anything new on a subject so nearly approaching perfection, and yet there are points in every one of these cases that will not fail to interest those who are working in this department of surgery.

There is no surgeon who does not learn something from his last operation. There is no collaborator who cannot extract something of profit from the work of others. This is my excuse for detaining you this evening. If I shall fail to weary you, and add ever so little of pleasure or profit to this meeting, I shall be amply repaid. I shall at once proceed to

COMPOUND UNILOCULAR OVARIAN TUMOR.

CASE I. Mrs. H., æt. about 56, was brought to the Hospital for the Women of Maryland in February, 1887, and placed under my care by Dr. George B. Reynolds. She had a very large tumor occupying nearly the whole abdominal cavity. It was diagnosed to be a compound multilocular tumor of the ovary. She was very feeble. The tumor had been growing for several years. She was very pale, had been badly nourished, lived in a very malarious part of the city, and most of her time was spent at the washtub in a damp, illy-lighted cellar.

She was put on quinine, and an attempt made to build up her health, but, instead of gaining, she was seen to be rapidly losing strength, and it was found necessary to operate at once or she would probably soon die of exhaustion.

On February 22, 1887, she was chloroformed by Dr. Wm. P. Chunn, and, assisted by Dr. Robert T. Wilson, in the presence of Dr. George B. Reynolds, I opened the abdomen. My diagnosis was confirmed. The walls of the tumor were so rotten that the gentlest manipulation ruptured them, and a dark, thick fluid was discharged into the abdominal cavity. I proceeded to separate adhesions, during which much of the rotten parts of the tumor were broken off and fell within the abdomen. The pedicle was transfixed with a needle and double ligature, which was cut and tied on either side, and the tumor was then cut away.

Several gallons of hot water were poured in and the abdominal cavity thoroughly washed out. The abdominal incision was closed with silk sutures, and no drainage-tube was used. The patient made a good recovery for ten days, when a typho-malarial fever developed, and for five weeks afterwards she was desperately ill. She finally got perfectly well, and is now in excellent health.

This case is of interest for the great amount of hot water found necessary to thoroughly cleanse the abdominal cavity and its contents, for the near approach to death before the operation from exhaustion, and for recovery, after weeks of severe illness, from a typho-malarial fever following close on such an operation.

PAPILLOMATOUS CYSTOMA OF THE OVARY.

CASE II. Miss H., æt. 45 years, consulted me for the first time September 10, 1887. She had always been a perfectly healthy woman till six weeks before; never sick in her life. She had menstruated regularly until the time of seeing me, when she had missed her menses for five days over her regular period.

Six weeks ago she observed her abdomen was enlarging, without appreciable ill-health. This enlargement increased rapidly, until when I saw her it was so distended with ascitic fluid, as to greatly impede her respiration. I could nowhere discover a tumor in the abdomen or pelvis, so great was the abdominal distension. The pelvis was free, only a moderate thickening of the right broad ligament. Uterus movable and normal in size, as shown by the probe. The great abdominal distension prevented the discovery of anything by bi-palpation.

The heart was sound, liver healthy; examination of urine revealed no disease of kidneys, urine scanty and full of urates; lungs sound, no anasarca.

Under these circumstances I diagnosed a papillomatous tumor of the ovary, and advised laparotomy, rather than tapping, for removal of the ascitic fluid, and, if possible, removal of the suspected tumor. I preferred cutting in to tapping, because I would then know the exact condition of things, and might be able at once to proceed to the removal of any tumor, which might be the cause of the ascites, and because if I were unable to remove the tumor, or might consider it unwise so to do, the abdomen was less likely to fill up rapidly again after an exploratory incision, than after tapping. This has been my experience in such cases. Moreover, I did not consider an incision much, if any, more dangerous than tapping. I left the city two days after this interview, and was absent one week.

I saw my patient again September 19. The abdominal enlargement was increasing rapidly. Dyspnœa was greater, and strength rapidly failing. Kidneys working badly. I again urged the operation, but the patient could not get her mind up to this point. She moreover plead the absence of members of her family, and was unwilling to consent to an operation till their return. I saw her every day or two, and urged the dangers of delay. In another week she became so exhausted as to be unable to move about, and had to rest in a semi-prone condition. Her family then returned, and Dr. William T. Howard was asked to see her with me, on October 3. He confirmed my plan of procedure, and an operation was appointed for Wednesday, October 5. The day before the operation her bowels gave way, and she had profuse diarrhœa, which was checked by liberal doses of paregoric. The night before the operation she slept soundly from the paregoric taken.

At 12 o'clock on October 5, being etherized by Dr. Wm. B. Canfield, and assisted by Dr. Wm. T. Howard and Dr. Robert T. Wilson, I cut into the abdominal cavity, making an incision of about two inches midway between the umbilicus and pelvis. Between two and three gallons of yellowish ascitic fluid was discharged. A ragged papillomatous mass presented, and on passing two fingers into the pelvis I found a large tumor growing from the fundus of the uterus and the right broad ligament and ovary. Masses of varied size broke away from the surface of the tumor on the most gentle manipulation, and bleeding was very free. With large compression-forceps I clamped the tumor close to the uterus and ovary and thus stopped hæmorrhage, having previously enlarged the incision to four inches. I then transfixed the broad pedicle with a needle, armed with a strong double ligature, which was cut and tied on either side, close to the uterus, and including the ovary. Each ligature was then brought entirely around the whole pedicle, and firmly tied again. The ends were cut short, and the tumor cut away.

Hot water was poured copiously into the abdominal cavity, and it was thus thoroughly washed out, much of the débris of the tumor coming away with the water. The peritoneum was in a state of sub-acute inflammation, with here and there a patch of recent lymph. A drainage-tube was inserted to the bottom of the pelvis, and the abdominal opening closed with twelve silk sutures.

The abdominal wound was dusted with iodoform—a linen cloth—absorbent cotton—a rubber cloth perforated by the drainage-tube, at the top of which was placed a sponge, and the rubber cloth folded over it, all secured by a flannel bandage. Hypodermics of whisky were necessary during the operation, which lasted over an hour.

The patient was then placed in bed, wrapped in warm blankets, with hot water bottles around her. Reaction came on very slowly.

At 10 p. m., temperature was 101° F., and pulse 140, and very feeble. No nausea. Ten drops of brandy in two teaspoonfuls of hot water were given every two hours. Ten drops of Magendie's solution of morphia were given hypodermically, and fifteen drops of tincture of digitalis were given every four hours, and continued for three days. The drainage-tube was sucked every two or three hours during the afternoon and night with a glass syringe and rubber tube attached. Much bloody, watery fluid was withdrawn, and occasionally a small clot of blood.

October 6, 8 a. m., temperature $102\frac{3}{5}$, pulse 136; slept some; very feeble. Ordered one teaspoonful of brandy, and three teaspoonfuls of hot water every two hours; one teaspoonful of lime water and two teaspoonfuls of milk every two hours. Digitalis as usual. Gave a hypodermic of 30 drops of muriate of quinia and urea. Drainage-tube sucked as yesterday, and half an ounce of bloody fluid withdrawn. None in the sponge. At 10 p. m., temperature and pulse same as in the morning. Thirty drops of quinia and urea were given as in the morning; digitalis continued; ten drops of Magendie's solution were given hypodermically at bedtime, but not repeated after to-day.

This treatment was continued for four days, except to increase the brandy to one tablespoonful every two hours, and the milk to eight tablespoonfuls every two hours. The highest temperature was $103\frac{4}{5}$, and for four days it varied from 101 to $103\frac{4}{5}$. Pulse was never under 120, and very feeble; the whole body was cool, and at times covered with a clammy sweat. Beef tea and brandy were injected into the rectum once or twice daily. Her prostration was very great.

At the end of the tenth day I removed the drainage-tube, although I was able to suck from it three or four times daily from two to four teaspoonfuls of a bloody, watery fluid. The tube was found to contain at its lower extremity a plug of solid lymph about one inch long.

October 13, one week after the operation. After the fourth day temperature fell below 100, and has not risen above it to this time. Pulse gradually subsided to 108, at which I found it to-day. Digitalis stopped after fourth day. Hypodermics of quinia stopped after eighth day. Her bowels were well moved by an injection of beef tea and brandy on the third day, and have been moved every day since. Passes flatus freely. Brandy and milk continued in same quantity, every two hours, night and day. Has some sero-purulent discharge from the seat of drainage-tube.

October 19, two weeks after the operation, temperature 98, pulse 100. Still some oozing of sero-purulent fluid from seat of drainage-tube. Removed all sutures twelve days after the operation. Union complete, except where the drainage-tube was. Tongue dry and red, complains of great weakness; bowels regular, and is much troubled with wind colic. Is taking large quantities of rich milk, with brandy or old port wine.

November 2, four weeks after the operation. Our patient has continued to improve slowly since last note. Has been eating and enjoying solid food for the past few days in addition to milk and brandy. Has been very nervous and depressed and sleepless. Opiates, bromides, and paraldehyde made her worse, and it was only when I put her on large doses of extract of sumbul and assafœtida in capsules that she became calm and cheerful, and obtained plenty of refreshing sleep. She has improved rapidly under these remedies, and sat up in a chair for the first time to-day.

November 8, convalescing rapidly. Has a good appetite; is cheerful and brighter, with no unpleasant symptom. Is sitting up and moving about her room. Drainage-tube opening healed.

Will this diseased mass, thoroughly removed from the pelvic organs of this lady, manifest its malignancy, by reappearing in the same place, or in some other part of her body? This is a question yet to be demonstrated.

The tumor was sent to Professor Wm. H. Welch of the Johns Hopkins University, for analysis. He returned to me the following report.

* * * "The growth is as you diagnosticated, a papillomatous cystoma of the ovary. I have repeatedly found in ovarian tumors, as in this case, cancerous nodules, which apparently produce no metastases, unless they grow through the external wall of the cyst.

"The specimen consists of only a part of the original tumor. The part removed measures 18 ctm. in diameter and 8 ctm. in depth.

"The tumor is a large cystoma, consisting apparently of a main cyst and numerous smaller cysts. The external cyst wall is smooth and glistening in most places, but there are the remnants of many old adhesions. There are also several irregular outgrowths from the exterior of the cyst. These outgrowths are solid and covered with the external layer of the cyst wall. The largest are about the size of a pigeon's egg.

"The interior of the cyst contains many papillomatous projections and much sloughy material. In the papillomata and in the main cyst wall are many cysts filled with thick, colloid substance.

"The pedicle consists of connective tissue and blood vessels, apparently belonging to the broad ligament. The fimbria ovarica is present, but nothing else belonging to the fallopian tube can be detected. There is a small parovarian cyst with clear contents near the attachment of the fimbria ovarica.

"Microscopical examination shows that the interior of the cysts is lined by cylindrical epithelium. The papillomata are covered with similar epithelium and contain alveolar spaces lined with such epithelium. There are solid masses in the cyst wall with the structure of carcinoma — alveoli filled with epithelial cells.

"Diagnosis: The tumor is an ovarian cystoma of the variety known as papillomatous. In addition to the ordinary appearance of such tumors, there are in the present instance on the cyst wall carcinomatous growths. The tumor is malignant.

"WM. H. WELCH."

### REMOVAL OF THE OVARIES AND TUBES AT THE PERIOD OF MENSTRUATION.

CASE III. Miss S., æt. 38, has been a patient of mine for about sixteen years. When she came under my care she had been a great sufferer for some time, confined to her bed, and having hystero-epileptiform convulsions, from five to ten per day, often of the most violent character. She had been unable to stand alone for months, and was brought to me on a bed from an adjoining State. She had terrible dismenorrhœa, and her whole nervous system was in a greatly demoralized state. I never encountered a patient who was a more complete wreck.

I found her with general pelvic cellulitis. Endometritis from cervix to fundus. Uterus anti-flexed and stenosed at the internal os. Her bowels were habitually, and necessarily, constipated. I have known her to go three weeks without an evacuation. At times it was impossible to move them with purgatives. Sometimes I had to rake out the rectum, and at other times I had to assist catharctics by passing a large and long gum tube up the bowels and throwing in the most active enemas.

By attending to the bowels, treating the cellulitis locally, and with constitutional means, she greatly improved; the convulsions ceased. She was able to walk about the city, and after several months under my care she was able to return home, a greatly changed woman. She has returned to me several times within the past sixteen years for treatment. Has as often been benefited, but never cured.

For the last few years her health has been declining. She has been again the subject of all sorts of reflected nervous symptoms, especially at the approach of each menstrual period, when she becomes at times melancholy, at other times greatly excited, so that she and her friends had grave apprehensions that she would lose her mind. Convulsions have recently returned at the time of menstruation, and she was becoming addicted to the use of anodynes.

Under these circumstances she returned again, appealing to me for relief; and on October 3, 1887, she was admitted to the Hospital for the Women of Maryland. A careful examination, with a knowledge of all that had been done for her in the past sixteen years, brought me to the conclusion that the removal of the ovaries and tubes held out to her the only hope for relief from her present suffering and probable future dementia.

After two weeks of preparatory treatment, with laxatives, tonics, and nervines, rest and recreation, so as to brace up a shattered nervous system, the 18th of October was appointed as the day for operating.

When I called to see her the evening before, she informed me that the 18th was the day when she should commence to menstruate, though she sometimes went a week or ten days over her regular time. She had misled me as to the time of expected menstruation, but as everything was now ready, as she was up to the operating point, as she could ill afford the expense of remaining long in the hospital, as I never consider it wise to postpone an operation when the day is once appointed, I told her that I would perform the operation on the following day, even if her menses did come on. She had been much alarmed by a meddlesome nurse telling her the great danger she would be in if she were operated on during menstruation, or if it came on immediately afterward. On the next day (October 18), she was etherized by Dr. Wm. P. Chunn; assisted by Dr. R. T. Wilson, I made an incision of about two inches into the abdominal cavity. The ovaries and tubes were found to be enlarged and bound down by old adhesions. The former were soft and friable, the latter were inflamed and tortuous, doubled up at some points upon themselves. The gentlest manipulation caused the ovaries to tear, and I had much difficulty in raising them sufficiently with the tubes to ligate them. A double ligature was passed beneath them, cut and tied on either side, and each ligature was again carried entirely around the pedicle and tied again. The ovaries and tubes were then cut away. There was some oozing from the late adhesions, but sponging was kept up till all was dry, and the abdominal opening was then closed with seven silk sutures, and the wound dressed with iodoform and a dry linen cloth secured by a bandage.

The patient did well. Her temperature rose one day to 101° F., and rapidly fell below 100.° Her pulse never exceeded 110 after reaction from the operation was established.

Menstruation came on the 20th, just two days after the operation, and disappeared n the 24th, without any rise of pulse or temperature.

A few hours after menstruation began her pulse was 98° and her temperature was $100\frac{2}{5}$.

She had less flow, and less nervous disturbance at this period than for many months before.

This is my third successful laparotomy done during, or followed by, menstruation within twenty-four or forty-eight hours. I have never lost a case operated on during this period, and I am beginning to think it is a propitious time for these operations. I never had a patient make a better recovery than this, or have less constitutional or local disturbance.

I herewith present to the Society the ovaries and tubes removed.

### A CASE OF NYMPHOMANIA.

Wm. Pawson Chunn, M. D., said: In the history of the case I wish to present, my efforts were exerted without avail, and it is mainly on that account, as well as to obtain the opinion of those present, that I recite the following symptoms: A short time since a patient from a neighboring State came under my care; she was in a deplorable condition, due to an ungovernable and continual desire for sexual intercourse. She was 23 years old, unmarried, and had never been pregnant. Her uterus was retroverted and hyperplastic. She complained of a numbness and pain in both ovarian regions, and had worn a pessary for a long time without relief either from the pain or the sexual desire; this last was her worst symptom. She deplored the fact that any one with sufficient opportunity could prevail over her scruples, as had already occurred several times. Sleep was lost at night, and rest disturbed by delusive dreams. She had never practiced any form of self-abuse. After a short course of the bromides, without effect, I suggested that marriage might give her relief. She objected to the motive; and, in addition, reminded me that she had derived no permanent benefit from illicit indulgence. Removal of the clitoris occurred to me, but only to be dismissed as unadvisable. She herself suggested the removal of the ovaries, as she felt satisfied that those organs must be diseased, thus accounting for the severe pelvic pain. This I considered to be too dangerous an experiment, as I had no certainty that she would be fully and permanently relieved by such an operation. It seemed to me that taking out the ovaries was not exactly analogous to removal of the testicles in the male. We know that castration of the male removes almost entirely all erotic desire, but the effect on the female has not been sufficiently investigated; about 15 per cent. of all women go through life without any sexual desire whatever, the removal of whose ovaries could have no effect. Desiring to ventilate this subject as fully as possible, I sought out several women who had, to my knowledge, lost their ovaries; one, upon whom the operation had been performed about five years since, told me that at present she had no erotic desire, but, at the same time she volunteered the information that she never had experienced any such desire. Two other women whom I next questioned answered similarly, much to my surprise. On the other hand I have now under my care two sisters, aged respectively 58 and 60 years, both of whom have long since passed the menopause, but each claims now as much sexual appetite as she possessed in youth. Inasmuch as the ovaries of these women must by this time have become atrophied from age, it is apparent that the ovaries are not the only sources of sexual desire. Bearing these facts in mind I was necessarily uncertain in regard to the prognosis in the case of my patient, and having told her as much, she sought the care of another physician.

### DISCUSSION.

Dr. G. Lane Taneyhill remarked that in 1866, while assistant physician at the Maryland Hospital for the Insane, he was in the habit of cauterizing the clitoris with a view of preventing the patient from indulging in masturbation; for nine-tenths of his cases of nymphomania were the result of masturbation; this habit being prevented while the organ was in a state of inflammation in consequence of the use of the caustic, he was the more able to control the nymphomania proper by the free use of elaterium and bromide of potassium and low diet. Cold lotions and cold baths did not avail in his cases of nymphomania as they did in cases of satyriasis. He was of the opinion that Dr. John Fonerden was not far out of the way when he so vigorously insisted that masturbation was closely allied with many forms of insanity, but in his (Dr. T.'s) experience among the insane he had found the disgusting habit as frequently a result, as a cause, of insanity, especially in cases of nymphomania..

### A NEW UTERINE CURETTE.

Dr. T. A. Ashby exhibited a new uterine curette.

The instrument is modeled after the plan of the scoop which is found on the end of the grooved director accompanying the majority of surgical-instrument cases. The scoop is attached to a long shaft and handle, so that it can be easily carried to the fundus of the uterus.

It is designed to occupy an intermediate position between Simon's sharp-cutting curette and Thomas' blunt curette. The advantages of the instrument are shown as follows:

1. The size of the instrument admits of its ready passage through the cervix without dilatation of the organ.

2. It is made of flexible metal, so that the shaft can be bent at any angle and thus admit of its application to the uterine cavity when the uterus is flexed upon itself.

3. It is severe enough in its action to cleanse the uterine mucous membrane of unhealthy granulations or vegetations without inflicting any injury upon normal tissue.

This curette will be found to do good service in cases where Thomas' blunt curette or Simon's sharp curette have been usually employed. It is more easy of introduction into the uterus than either of these instruments, and can be employed with equal advantage and greater facility.

